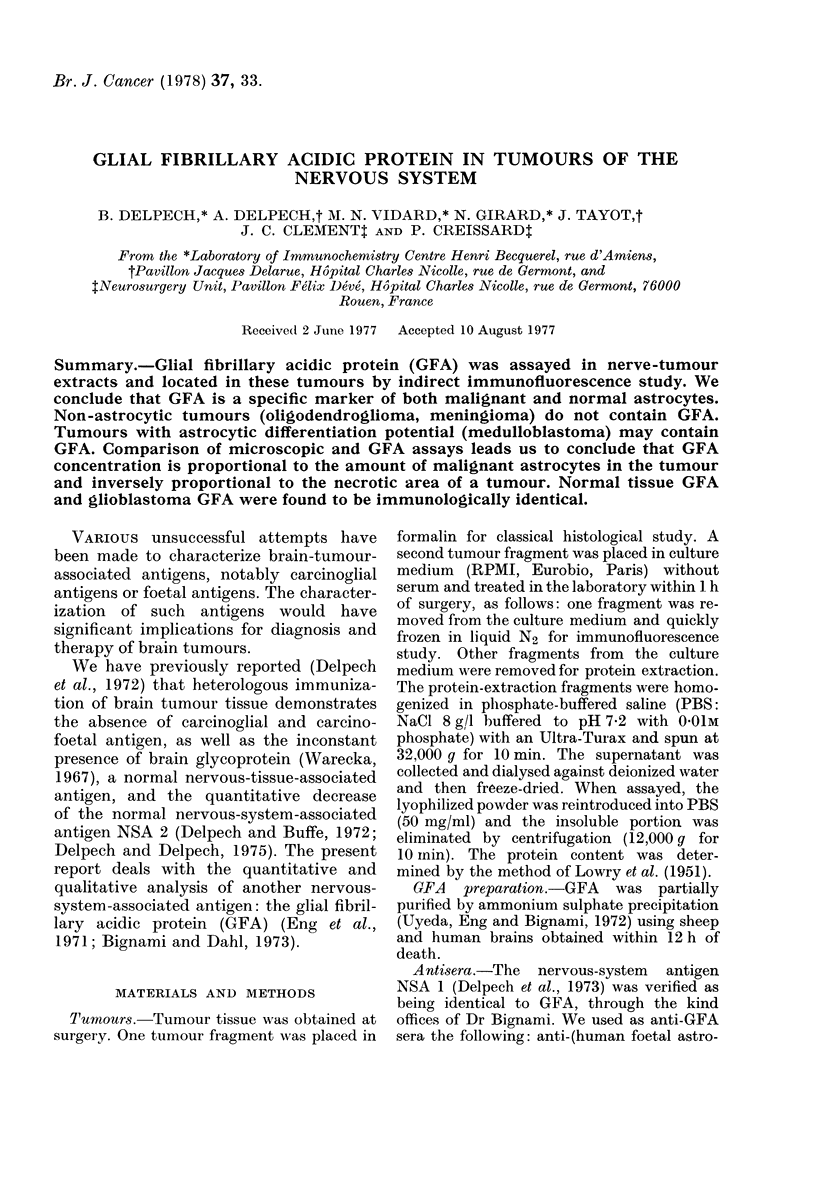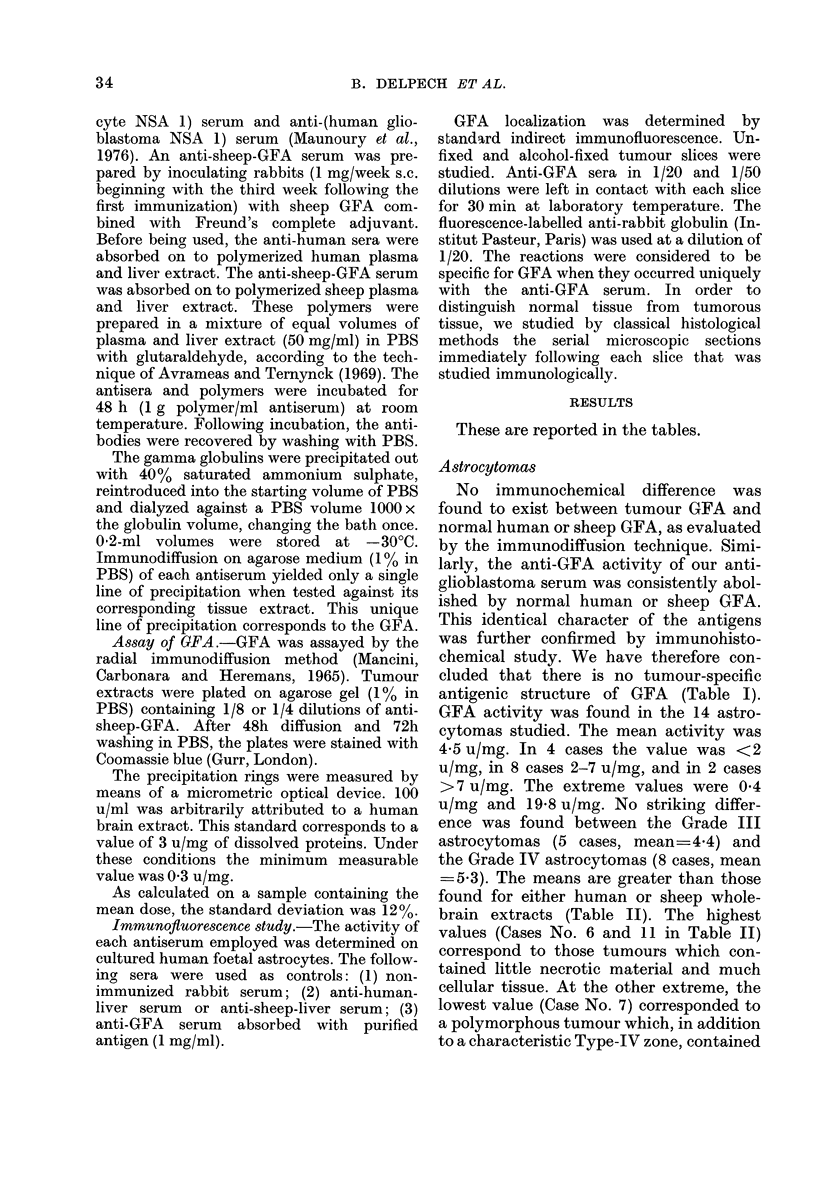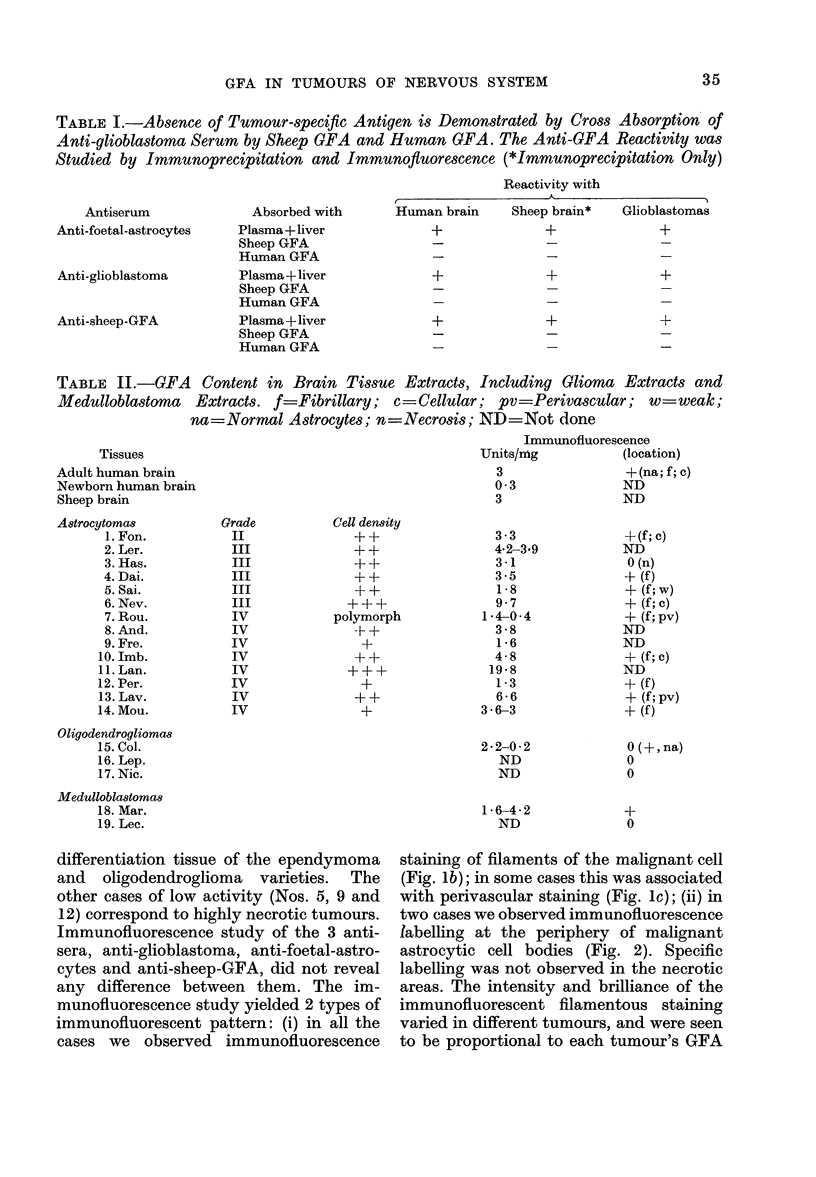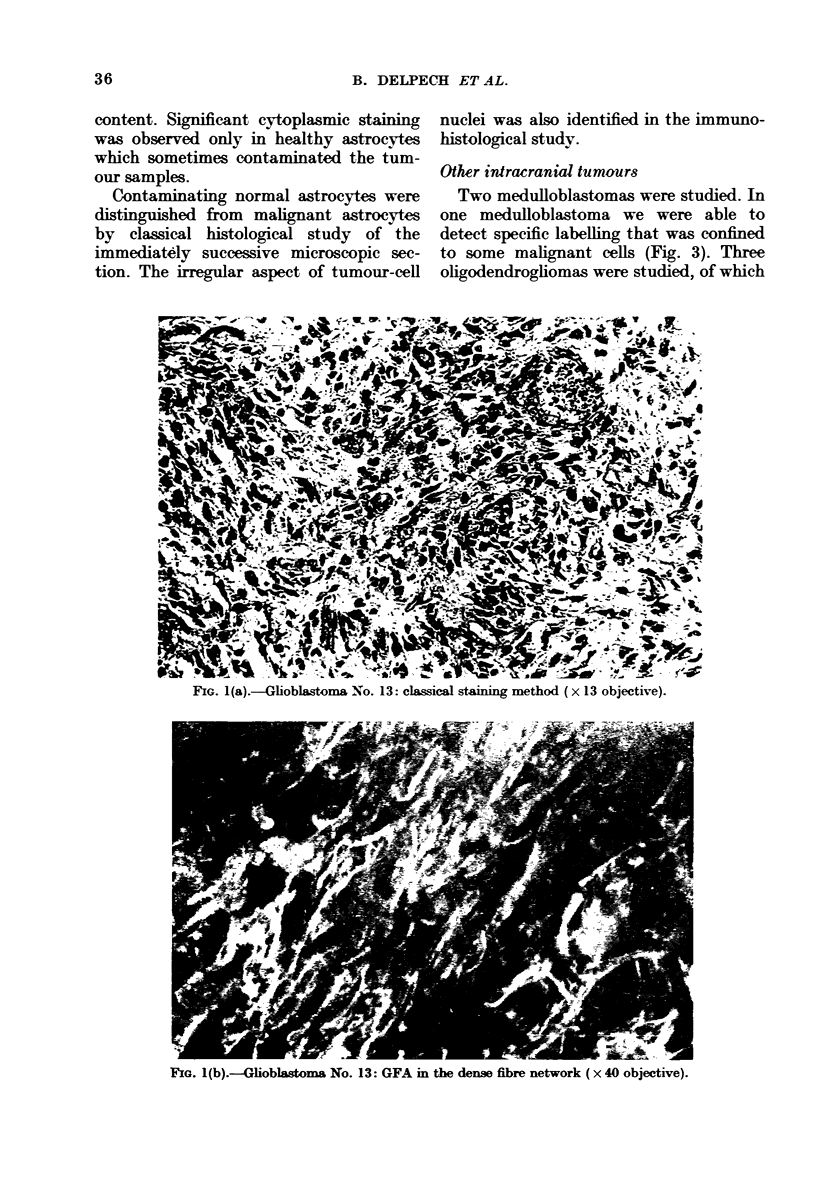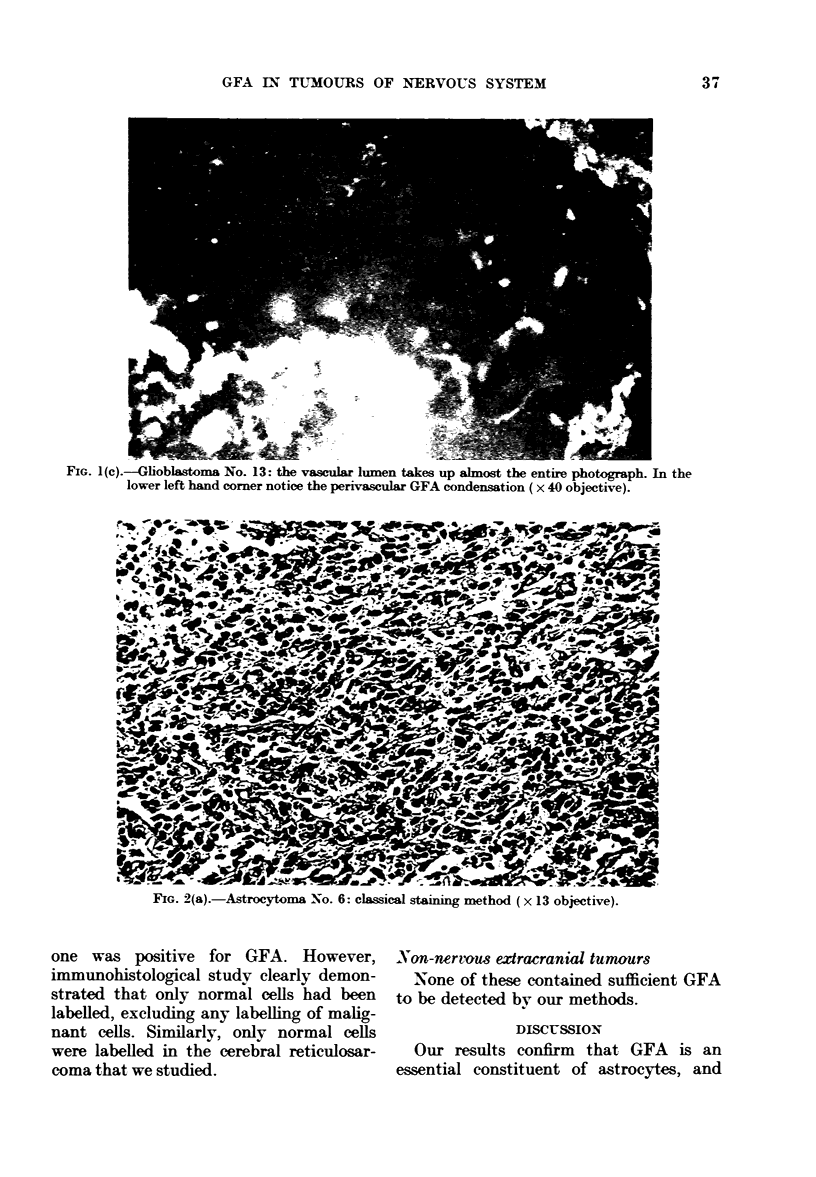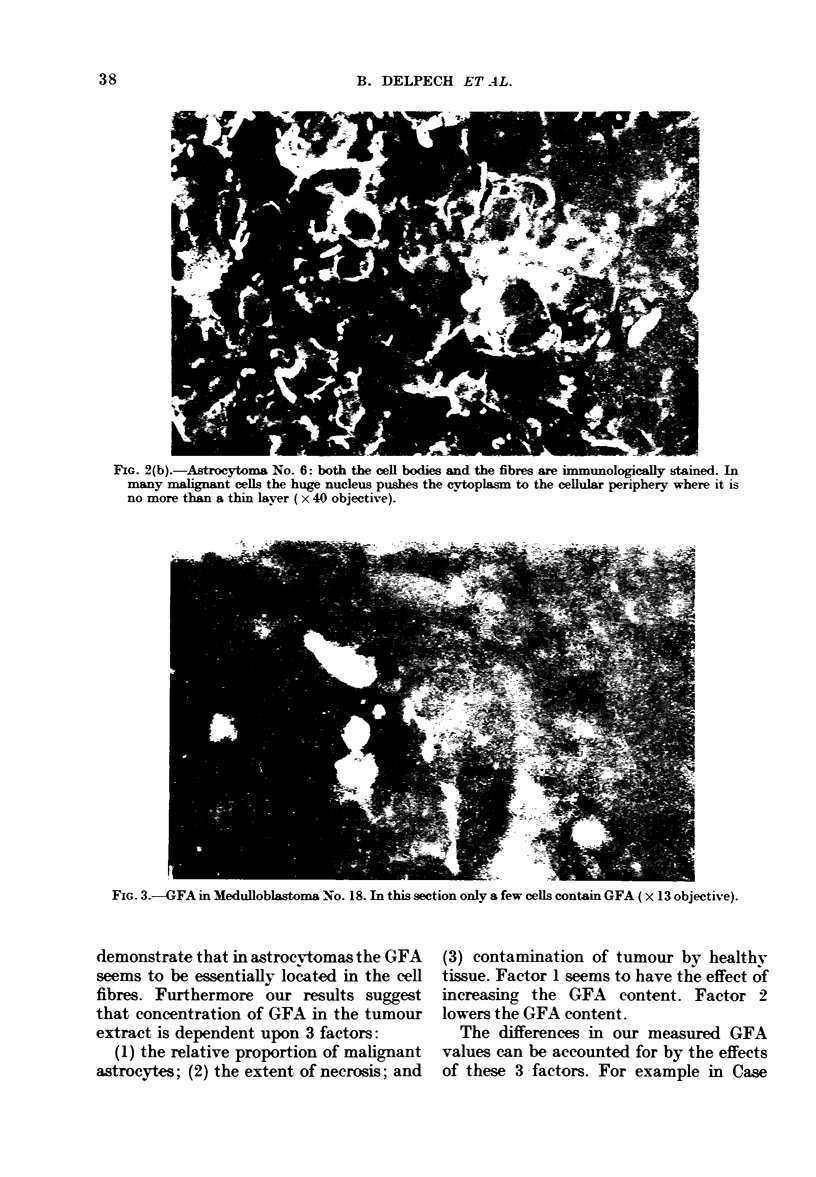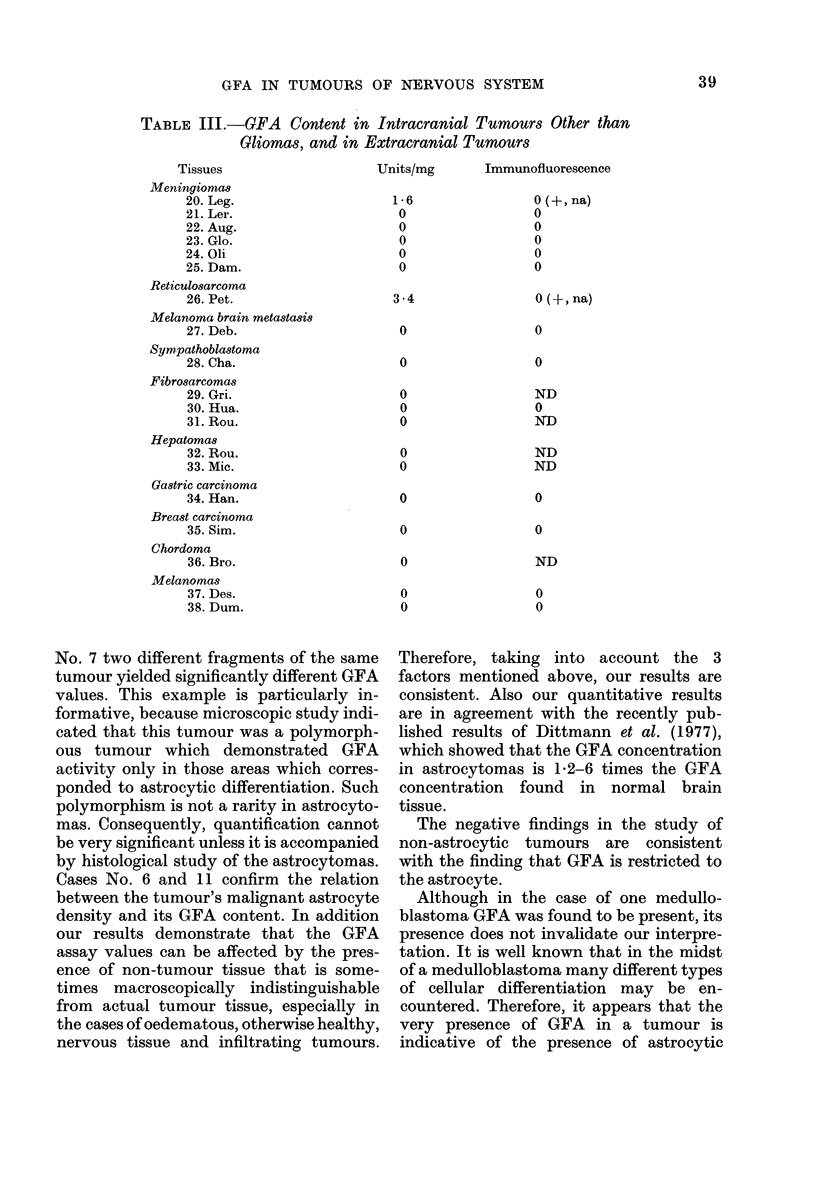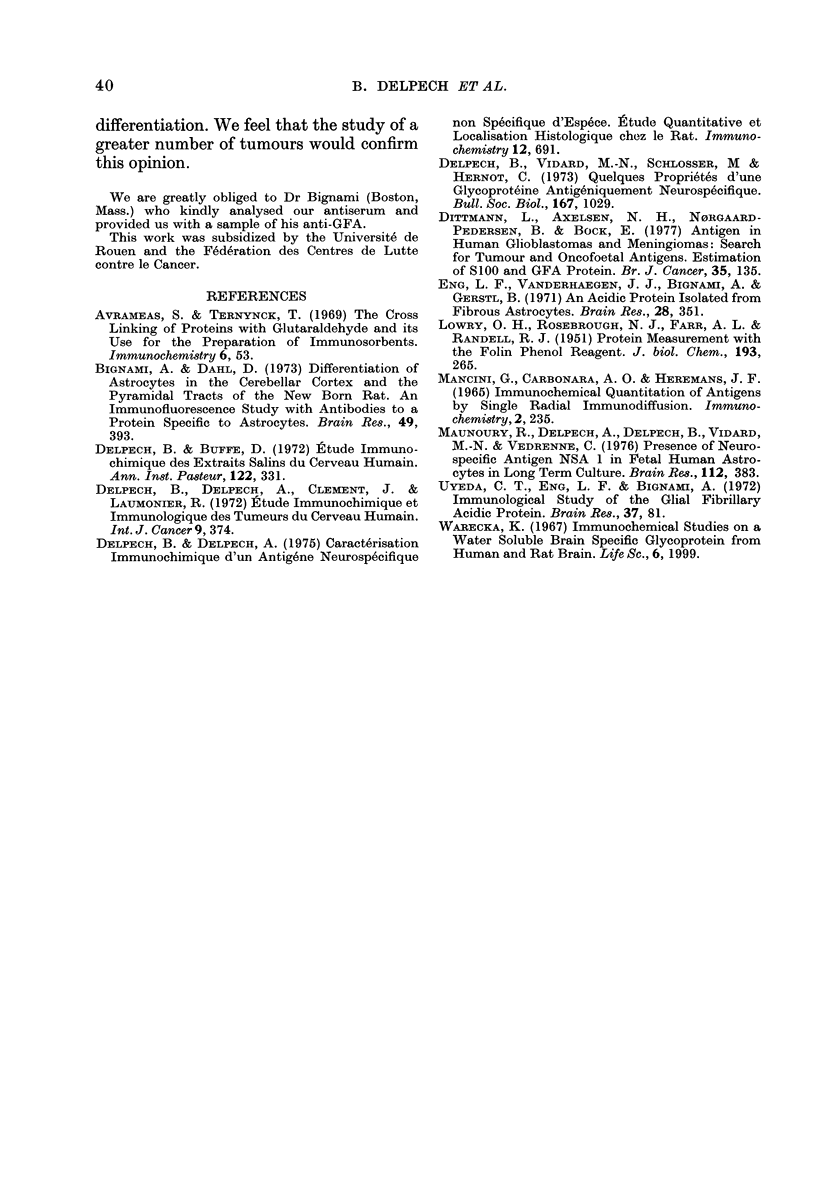# Glial fibrillary acidic protein in tumours of the nervous system.

**DOI:** 10.1038/bjc.1978.5

**Published:** 1978-01

**Authors:** B. Delpech, A. Delpech, M. N. Vidard, N. Girard, J. Tayot, J. C. Clement, P. Creissard

## Abstract

**Images:**


*Br. J. Cancer* (1978) **37**, 33.

### GLIAL FIBRILLARY ACIDIC PROTEIN IN TUMOURS OF THE NERVOUS SYSTEM

B. DELPECH,* A. DELPECH,† M. N. VIDARD,* N. GIRARD,* J. TAYOT,† J. C. CLEMENT‡ AND P. CREISSARD‡

*From the *Laboratory of Immunochemistry Centre Henri Becquerel, rue d'Amiens, †Pavillon Jacques Delarue, Hôpital Charles Nicolle, rue de Germont, and ‡Neurosurgery Unit, Pavillon Félix Dévé, Hôpital Charles Nicolle, rue de Germont, 76000 Rouen, France*

Received 2 June 1977  Accepted 10 August 1977

**Summary.—Glial fibrillary acidic protein (GFA) was assayed in nerve-tumour extracts and located in these tumours by indirect immunofluorescence study. We conclude that GFA is a specific marker of both malignant and normal astrocytes. Non-astrocytic tumours (oligodendroglioma, meningioma) do not contain GFA. Tumours with astrocytic differentiation potential (medulloblastoma) may contain GFA. Comparison of microscopic and GFA assays leads us to conclude that GFA concentration is proportional to the amount of malignant astrocytes in the tumour and inversely proportional to the necrotic area of a tumour. Normal tissue GFA and glioblastoma GFA were found to be immunologically identical.**

Various unsuccessful attempts have been made to characterize brain-tumour-associated antigens, notably carcinoglial antigens or foetal antigens. The characterization of such antigens would have significant implications for diagnosis and therapy of brain tumours.

We have previously reported (Delpech *et al.*, 1972) that heterologous immunization of brain tumour tissue demonstrates the absence of carcinoglial and carcino-foetal antigen, as well as the inconstant presence of brain glycoprotein (Warecka, 1967), a normal nervous-tissue-associated antigen, and the quantitative decrease of the normal nervous-system-associated antigen NSA 2 (Delpech and Buffe, 1972; Delpech and Delpech, 1975). The present report deals with the quantitative and qualitative analysis of another nervous-system-associated antigen: the glial fibrillary acidic protein (GFA) (Eng *et al.*, 1971; Bignami and Dahl, 1973).

### MATERIALS AND METHODS

*Tumours.*—Tumour tissue was obtained at surgery. One tumour fragment was placed in formalin for classical histological study. A second tumour fragment was placed in culture medium (RPMI, Eurobio, Paris) without serum and treated in the laboratory within 1 h of surgery, as follows: one fragment was removed from the culture medium and quickly frozen in liquid $N_2$ for immunofluorescence study. Other fragments from the culture medium were removed for protein extraction. The protein-extraction fragments were homogenized in phosphate-buffered saline (PBS: NaCl 8 g/l buffered to pH 7·2 with 0·01M phosphate) with an Ultra-Turax and spun at 32,000 *g* for 10 min. The supernatant was collected and dialysed against deionized water and then freeze-dried. When assayed, the lyophilized powder was reintroduced into PBS (50 mg/ml) and the insoluble portion was eliminated by centrifugation (12,000 *g* for 10 min). The protein content was determined by the method of Lowry *et al.* (1951).

*GFA preparation.*—GFA was partially purified by ammonium sulphate precipitation (Uyeda, Eng and Bignami, 1972) using sheep and human brains obtained within 12 h of death.

*Antisera.*—The nervous-system antigen NSA 1 (Delpech *et al.*, 1973) was verified as being identical to GFA, through the kind offices of Dr Bignami. We used as anti-GFA sera the following: anti-(human foetal astrocyte NSA 1) serum and anti-(human glioblastoma NSA 1) serum (Maunoury *et al.*, 1976). An anti-sheep-GFA serum was prepared by inoculating rabbits (1 mg/week s.c. beginning with the third week following the first immunization) with sheep GFA combined with Freund's complete adjuvant. Before being used, the anti-human sera were absorbed on to polymerized human plasma and liver extract. The anti-sheep-GFA serum was absorbed on to polymerized sheep plasma and liver extract. These polymers were prepared in a mixture of equal volumes of plasma and liver extract (50 mg/ml) in PBS with glutaraldehyde, according to the technique of Avrameas and Ternynck (1969). The antisera and polymers were incubated for 48 h (1 g polymer/ml antiserum) at room temperature. Following incubation, the antibodies were recovered by washing with PBS.

The gamma globulins were precipitated out with 40% saturated ammonium sulphate, reintroduced into the starting volume of PBS and dialyzed against a PBS volume 1000× the globulin volume, changing the bath once. 0·2-ml volumes were stored at −30°C. Immunodiffusion on agarose medium (1% in PBS) of each antiserum yielded only a single line of precipitation when tested against its corresponding tissue extract. This unique line of precipitation corresponds to the GFA.

*Assay of GFA.*—GFA was assayed by the radial immunodiffusion method (Mancini, Carbonara and Heremans, 1965). Tumour extracts were plated on agarose gel (1% in PBS) containing 1/8 or 1/4 dilutions of anti-sheep-GFA. After 48h diffusion and 72h washing in PBS, the plates were stained with Coomassie blue (Gurr, London).

The precipitation rings were measured by means of a micrometric optical device. 100 u/ml was arbitrarily attributed to a human brain extract. This standard corresponds to a value of 3 u/mg of dissolved proteins. Under these conditions the minimum measurable value was 0·3 u/mg.

As calculated on a sample containing the mean dose, the standard deviation was 12%.

*Immunofluorescence study.*—The activity of each antiserum employed was determined on cultured human foetal astrocytes. The following sera were used as controls: (1) non-immunized rabbit serum; (2) anti-human-liver serum or anti-sheep-liver serum; (3) anti-GFA serum absorbed with purified antigen (1 mg/ml).

GFA localization was determined by standard indirect immunofluorescence. Unfixed and alcohol-fixed tumour slices were studied. Anti-GFA sera in 1/20 and 1/50 dilutions were left in contact with each slice for 30 min at laboratory temperature. The fluorescence-labelled anti-rabbit globulin (Institut Pasteur, Paris) was used at a dilution of 1/20. The reactions were considered to be specific for GFA when they occurred uniquely with the anti-GFA serum. In order to distinguish normal tissue from tumorous tissue, we studied by classical histological methods the serial microscopic sections immediately following each slice that was studied immunologically.

### RESULTS

These are reported in the tables.

### *Astrocytomas*

No immunochemical difference was found to exist between tumour GFA and normal human or sheep GFA, as evaluated by the immunodiffusion technique. Similarly, the anti-GFA activity of our anti-glioblastoma serum was consistently abolished by normal human or sheep GFA. This identical character of the antigens was further confirmed by immunohistochemical study. We have therefore concluded that there is no tumour-specific antigenic structure of GFA (Table I). GFA activity was found in the 14 astrocytomas studied. The mean activity was 4·5 u/mg. In 4 cases the value was <2 u/mg, in 8 cases 2–7 u/mg, and in 2 cases >7 u/mg. The extreme values were 0·4 u/mg and 19·8 u/mg. No striking difference was found between the Grade III astrocytomas (5 cases, mean=4·4) and the Grade IV astrocytomas (8 cases, mean =5·3). The means are greater than those found for either human or sheep whole-brain extracts (Table II). The highest values (Cases No. 6 and 11 in Table II) correspond to those tumours which contained little necrotic material and much cellular tissue. At the other extreme, the lowest value (Case No. 7) corresponded to a polymorphous tumour which, in addition to a characteristic Type-IV zone, contained

TABLE I.—*Absence of Tumour-specific Antigen is Demonstrated by Cross Absorption of Anti-glioblastoma Serum by Sheep GFA and Human GFA. The Anti-GFA Reactivity was Studied by Immunoprecipitation and Immunofluorescence (*Immunoprecipitation Only)*

| | | Reactivity with | | |
|---|---|---|---|---|
| Antiserum | Absorbed with | Human brain | Sheep brain* | Glioblastomas |
| Anti-foetal-astrocytes | Plasma+liver | + | + | + |
| | Sheep GFA | − | − | − |
| | Human GFA | − | − | − |
| Anti-glioblastoma | Plasma+liver | + | + | + |
| | Sheep GFA | − | − | − |
| | Human GFA | − | − | − |
| Anti-sheep-GFA | Plasma+liver | + | + | + |
| | Sheep GFA | − | − | − |
| | Human GFA | − | − | − |

TABLE II.—*GFA Content in Brain Tissue Extracts, Including Glioma Extracts and Medulloblastoma Extracts. f=Fibrillary; c=Cellular; pv=Perivascular; w=weak; na=Normal Astrocytes; n=Necrosis;* ND=Not done

| Tissues | | | Units/mg | Immunofluorescence (location) |
|---|---|---|---|---|
| Adult human brain | | | 3 | +(na; f; c) |
| Newborn human brain | | | 0·3 | ND |
| Sheep brain | | | 3 | ND |
| *Astrocytomas* | *Grade* | *Cell density* | | |
| 1. Fon. | II | ++ | 3·3 | +(f; c) |
| 2. Ler. | III | ++ | 4·2–3·9 | ND |
| 3. Has. | III | ++ | 3·1 | 0 (n) |
| 4. Dai. | III | ++ | 3·5 | + (f) |
| 5. Sai. | III | ++ | 1·8 | + (f; w) |
| 6. Nev. | III | +++ | 9·7 | + (f; c) |
| 7. Rou. | IV | polymorph | 1·4–0·4 | + (f; pv) |
| 8. And. | IV | ++ | 3·8 | ND |
| 9. Fre. | IV | + | 1·6 | ND |
| 10. Imb. | IV | ++ | 4·8 | + (f; c) |
| 11. Lan. | IV | +++ | 19·8 | ND |
| 12. Per. | IV | + | 1·3 | + (f) |
| 13. Lav. | IV | ++ | 6·6 | + (f; pv) |
| 14. Mou. | IV | + | 3·6–3 | + (f) |
| *Oligodendrogliomas* | | | | |
| 15. Col. | | | 2·2–0·2 | 0 (+, na) |
| 16. Lep. | | | ND | 0 |
| 17. Nic. | | | ND | 0 |
| *Medulloblastomas* | | | | |
| 18. Mar. | | | 1·6–4·2 | + |
| 19. Lec. | | | ND | 0 |

differentiation tissue of the ependymoma and oligodendroglioma varieties. The other cases of low activity (Nos. 5, 9 and 12) correspond to highly necrotic tumours. Immunofluorescence study of the 3 antisera, anti-glioblastoma, anti-foetal-astrocytes and anti-sheep-GFA, did not reveal any difference between them. The immunofluorescence study yielded 2 types of immunofluorescent pattern: (i) in all the cases we observed immunofluorescence staining of filaments of the malignant cell (Fig. 1*b*); in some cases this was associated with perivascular staining (Fig. 1*c*); (ii) in two cases we observed immunofluorescence labelling at the periphery of malignant astrocytic cell bodies (Fig. 2). Specific labelling was not observed in the necrotic areas. The intensity and brilliance of the immunofluorescent filamentous staining varied in different tumours, and were seen to be proportional to each tumour's GFA

content. Significant cytoplasmic staining was observed only in healthy astrocytes which sometimes contaminated the tumour samples.

Contaminating normal astrocytes were distinguished from malignant astrocytes by classical histological study of the immediately successive microscopic section. The irregular aspect of tumour-cell nuclei was also identified in the immunohistological study.

*Other intracranial tumours*

Two medulloblastomas were studied. In one medulloblastoma we were able to detect specific labelling that was confined to some malignant cells (Fig. 3). Three oligodendrogliomas were studied, of which

FIG. 1(a).—Glioblastoma No. 13: classical staining method (×13 objective).

FIG. 1(b).—Glioblastoma No. 13: GFA in the dense fibre network (×40 objective).

FIG. 1(c).—Glioblastoma No. 13: the vascular lumen takes up almost the entire photograph. In the lower left hand corner notice the perivascular GFA condensation ( × 40 objective).

FIG. 2(a).—Astrocytoma No. 6: classical staining method ( × 13 objective).

one was positive for GFA. However, immunohistological study clearly demonstrated that only normal cells had been labelled, excluding any labelling of malignant cells. Similarly, only normal cells were labelled in the cerebral reticulosarcoma that we studied.

*Non-nervous extracranial tumours*

None of these contained sufficient GFA to be detected by our methods.

### DISCUSSION

Our results confirm that GFA is an essential constituent of astrocytes, and

FIG. 2(b).—Astrocytoma No. 6: both the cell bodies and the fibres are immunologically stained. In many malignant cells the huge nucleus pushes the cytoplasm to the cellular periphery where it is no more than a thin layer ( × 40 objective).

FIG. 3.—GFA in Medulloblastoma No. 18. In this section only a few cells contain GFA ( × 13 objective).

demonstrate that in astrocytomas the GFA seems to be essentially located in the cell fibres. Furthermore our results suggest that concentration of GFA in the tumour extract is dependent upon 3 factors:

(1) the relative proportion of malignant astrocytes; (2) the extent of necrosis; and (3) contamination of tumour by healthy tissue. Factor 1 seems to have the effect of increasing the GFA content. Factor 2 lowers the GFA content.

The differences in our measured GFA values can be accounted for by the effects of these 3 factors. For example in Case

TABLE III.—*GFA Content in Intracranial Tumours Other than Gliomas, and in Extracranial Tumours*

| Tissues | Units/mg | Immunofluorescence |
|---|---|---|
| *Meningiomas* | | |
| 20. Leg. | 1·6 | 0 (+, na) |
| 21. Ler. | 0 | 0 |
| 22. Aug. | 0 | 0 |
| 23. Glo. | 0 | 0 |
| 24. Oli | 0 | 0 |
| 25. Dam. | 0 | 0 |
| *Reticulosarcoma* | | |
| 26. Pet. | 3·4 | 0 (+, na) |
| *Melanoma brain metastasis* | | |
| 27. Deb. | 0 | 0 |
| *Sympathoblastoma* | | |
| 28. Cha. | 0 | 0 |
| *Fibrosarcomas* | | |
| 29. Gri. | 0 | ND |
| 30. Hua. | 0 | 0 |
| 31. Rou. | 0 | ND |
| *Hepatomas* | | |
| 32. Rou. | 0 | ND |
| 33. Mic. | 0 | ND |
| *Gastric carcinoma* | | |
| 34. Han. | 0 | 0 |
| *Breast carcinoma* | | |
| 35. Sim. | 0 | 0 |
| *Chordoma* | | |
| 36. Bro. | 0 | ND |
| *Melanomas* | | |
| 37. Des. | 0 | 0 |
| 38. Dum. | 0 | 0 |

No. 7 two different fragments of the same tumour yielded significantly different GFA values. This example is particularly informative, because microscopic study indicated that this tumour was a polymorphous tumour which demonstrated GFA activity only in those areas which corresponded to astrocytic differentiation. Such polymorphism is not a rarity in astrocytomas. Consequently, quantification cannot be very significant unless it is accompanied by histological study of the astrocytomas. Cases No. 6 and 11 confirm the relation between the tumour's malignant astrocyte density and its GFA content. In addition our results demonstrate that the GFA assay values can be affected by the presence of non-tumour tissue that is sometimes macroscopically indistinguishable from actual tumour tissue, especially in the cases of oedematous, otherwise healthy, nervous tissue and infiltrating tumours. Therefore, taking into account the 3 factors mentioned above, our results are consistent. Also our quantitative results are in agreement with the recently published results of Dittmann *et al.* (1977), which showed that the GFA concentration in astrocytomas is 1·2–6 times the GFA concentration found in normal brain tissue.

The negative findings in the study of non-astrocytic tumours are consistent with the finding that GFA is restricted to the astrocyte.

Although in the case of one medulloblastoma GFA was found to be present, its presence does not invalidate our interpretation. It is well known that in the midst of a medulloblastoma many different types of cellular differentiation may be encountered. Therefore, it appears that the very presence of GFA in a tumour is indicative of the presence of astrocytic differentiation. We feel that the study of a greater number of tumours would confirm this opinion.

We are greatly obliged to Dr Bignami (Boston, Mass.) who kindly analysed our antiserum and provided us with a sample of his anti-GFA.

This work was subsidized by the Université de Rouen and the Fédération des Centres de Lutte contre le Cancer.

### REFERENCES

AVRAMEAS, S. & TERNYNCK, T. (1969) The Cross Linking of Proteins with Glutaraldehyde and its Use for the Preparation of Immunosorbents. *Immunochemistry* **6**, 53.

BIGNAMI, A. & DAHL, D. (1973) Differentiation of Astrocytes in the Cerebellar Cortex and the Pyramidal Tracts of the New Born Rat. An Immunofluorescence Study with Antibodies to a Protein Specific to Astrocytes. *Brain Res.*, **49**, 393.

DELPECH, B. & BUFFE, D. (1972) Étude Immunochimique des Extraits Salins du Cerveau Humain. *Ann. Inst. Pasteur*, **122**, 331.

DELPECH, B., DELPECH, A., CLEMENT, J. & LAUMONIER, R. (1972) Étude Immunochimique et Immunologique des Tumeurs du Cerveau Humain. *Int. J. Cancer* **9**, 374.

DELPECH, B. & DELPECH, A. (1975) Caractérisation Immunochimique d'un Antigéne Neurospécifique non Spécifique d'Espéce. Étude Quantitative et Localisation Histologique chez le Rat. *Immunochemistry* **12**, 691.

DELPECH, B., VIDARD, M.-N., SCHLOSSER, M & HERNOT, C. (1973) Quelques Propriétés d'une Glycoprotéine Antigéniquement Neurospécifique. *Bull. Soc. Biol.*, **167**, 1029.

DITTMANN, L., AXELSEN, N. H., NØRGAARD-PEDERSEN, B. & BOCK, E. (1977) Antigen in Human Glioblastomas and Meningiomas: Search for Tumour and Oncofoetal Antigens. Estimation of S100 and GFA Protein. *Br. J. Cancer*, **35**, 135.

ENG, L. F., VANDERHAEGEN, J. J., BIGNAMI, A. & GERSTL, B. (1971) An Acidic Protein Isolated from Fibrous Astrocytes. *Brain Res.*, **28**, 351.

LOWRY, O. H., ROSEBROUGH, N. J., FARR, A. L. & RANDELL, R. J. (1951) Protein Measurement with the Folin Phenol Reagent. *J. biol. Chem.*, **193**, 265.

MANCINI, G., CARBONARA, A. O. & HEREMANS, J. F. (1965) Immunochemical Quantitation of Antigens by Single Radial Immunodiffusion. *Immunochemistry*, **2**, 235.

MAUNOURY, R., DELPECH, A., DELPECH, B., VIDARD, M.-N. & VEDRENNE, C. (1976) Presence of Neurospecific Antigen NSA 1 in Fetal Human Astrocytes in Long Term Culture. *Brain Res.*, **112**, 383.

UYEDA, C. T., ENG, L. F. & BIGNAMI, A. (1972) Immunological Study of the Glial Fibrillary Acidic Protein. *Brain Res.*, **37**, 81.

WARECKA, K. (1967) Immunochemical Studies on a Water Soluble Brain Specific Glycoprotein from Human and Rat Brain. *Life Sc.*, **6**, 1999.